# Clinical usefulness of serial neutrophil geletinaseassociated lipocalin (NGAL) change as a predictor for acute kidney injury

**DOI:** 10.1186/2197-425X-3-S1-A260

**Published:** 2015-10-01

**Authors:** H Yu, S Park, Y Choe, S Kim, S Park, Y Lee, H Lee

**Affiliations:** Chonbuk National University Hospital, Nursing, Jeonju, Korea, Republic of Korea; Chonbuk National University Hospital, Internal Medicine, Jeonju, Korea, Republic of Korea

## Intr

Plasma neutrophil geletinase-associated lipocalin (NGAL) has been regarded as one of the valuable markers of development of acute kidney injury (AKI) in ICU admitted patients. However, clinical significance of short-term change of plasma NGAL was not clearly investigated.

## Methods

50 patients who admitted intensive care unit (ICU) was included by prospective manner. We analyzed plasma NGAL level by Triage immunoassay on ICU admitted time and 12 hours after. Change of estimated glomerular filtration rate (by MDRD equation) and need of renal replacement therapy was investigated. Patients with chronic kidney disease were excluded.

## Results

Persistent elevation of plasma NGAL above 1000 ng/mL on 0 and 12 hours was significantly associated with need for early renal replacement therapy. Patient with increased level of NGAL at 12 hours showed unfavorable outcome which is related to renal replacement therapy or progressive renal impairment (13/25; 48% of patients). Conversely, 2/17 (11.8%) of patients had poor renal outcome when NGAL decrease more than 20% from baseline at 12 hours after ICU admission. On multivariate analysis, baseline NGAL and ΔNGAL_0-12 hr_ was independent predictors of unfavorable renal outcome. However, ΔNGAL_0-12hr_ did not significantly correlated with the change of glomerular filtration rate between day 1 and day3.

## Conclusions

Our data suggest that measurement of early serum NGAL change may be useful marker in predicting the renal impairment and need for renal replacement therapy in intensive care unit.

